# High SARS-CoV-2 seroincidence but low excess COVID mortality in Sierra Leone in 2020–2022

**DOI:** 10.1371/journal.pgph.0003411

**Published:** 2024-09-10

**Authors:** Ahmed Osman, Ashley Aimone, Rashid Ansumana, Isaac Bogoch, Hellen Gelband, Karen Colwill, Anne-Claude Gingras, Marc-André Langlois, Ronald Carshon-Marsh, Ibrahim Bob Swaray, Amara Jambai, Mohamed Vandi, Alimatu Vandi, Mohamed Massaquoi, Anteneh Assalif, H. Chaim Birnboim, Patrick E. Brown, Nico Nagelkerke, Prabhat Jha

**Affiliations:** 1 Centre for Global Health Research, Unity Health Toronto and Dalla Lana School of Public Health, University of Toronto, Toronto, Ontario, Canada; 2 School of Community Health Sciences Njala University, Bo, Sierra Leone; 3 Department of Medicine, University of Toronto, Toronto, Ontario, Canada; 4 Lunenfeld-Tanenbaum Research Institute, Sinai Health, Toronto, Ontario, Canada; 5 Department of Biochemistry, Microbiology and Immunology, University of Ottawa, Ottawa, Ontario, Canada; 6 Ministry of Health, Government of Sierra Leone, Freetown, Sierra Leone; 7 National Civil Registration Authority, Freetown, Sierra Leone; 8 deltaDNA Biosciences, Inc, Toronto, Ontario, Canada; University of Hong Kong, HONG KONG

## Abstract

While SARS-CoV-2 infection appears to have spread widely throughout Africa, documentation of associated mortality is limited. We implemented a representative serosurvey in one city of Sierra Leone in Western Africa, paired with nationally representative mortality and selected death registration data. Cumulative seroincidence using high quality SARS-CoV-2 serological assays was 69% by July 2021, rising to 84% by April 2022, mostly preceding SARS-CoV-2 vaccination. About half of infections showed evidence of neutralizing antibodies. However, excess death rates were low, and were concentrated at older ages. During the peak weeks of viral activity, excess mortality rates were 22% for individuals aged 30–69 years and 70% for those over 70. Based on electronic verbal autopsy with dual independent physician assignment of causes, excess deaths during viral peaks from respiratory infections were notable. Excess deaths differed little across specific causes that, a priori, are associated with COVID, and the pattern was consistent among adults with or without chronic disease risk factors. The overall 6% excess of deaths at ages ≥30 from 2020–2022 in Sierra Leone is markedly lower than reported from South Africa, India, and Latin America. Thus, while SARS-CoV-2 infection was widespread, our study highlights as yet unidentified mechanisms of heterogeneity in susceptibility to severe disease in parts of Africa.

## Introduction

Cumulative SARS-CoV-2 seroincidence rose rapidly in sub-Saharan African (SSA) countries, from below 5% in June 2020 to nearly 90% by December 2021, according to a meta-analyses of 47 serostudies in 23 countries [1]. This increase occurred before widespread SARS-CoV-2 vaccination in Africa and before the worldwide emergence of the Omicron variant in late 2021/early 2022 (https://ourworldindata.org/coronavirus). The course, severity, and infection-fatality rate of SARS-CoV-2 infections in SSA remain largely unexplored as routine registration of deaths and medical certification of their causes remain uncommon in most of Africa [2].

The World Health Organization (WHO), using data derived from other countries, estimated 1.25 million excess deaths in Africa (1 million outside of South Africa) during viral waves over the two years of 2020–2021, including nearly 8,000 [2] in Sierra Leone (with lower and upper bounds of 480 and 15,700 deaths, respectively) [2]. Sierra Leone had a population of 8.3 million in 2022, of whom over 40% lived in urban areas, including over one million in the capital of Freetown. Sierra Leone had four major viral waves of SARS-CoV-2, during much the same weeks as observed in South Africa and much of the rest of the continent. The 7,762 officially confirmed COVID cases and 125 deaths since March 2020 are widely regarded as underestimates. Sierra Leone adopted stringent public health lock downs and restrictions during the initial wave in March-June 2020, but subsequent restrictions were far lower [3]. Complete vaccine (two-dose) coverage reached only 5% of Sierra Leoneans by December 2021, rising to 42% by December 2022 (https://ourworldindata.org/coronavirus).

We have assessed cumulative SARS-CoV-2 seroincidence in a representative sample of urban Bo, the second most populous city in Sierra Leone, using high-quality antibody assays [4]. We further examined excess and cause-specific mortality using nationally representative mortality data from an ongoing large mortality study covering a randomly selected 5% of Sierra Leone’s population [5], and non-random death registration data covering approximately 25% of the population.

## Results

**Fig 1:**
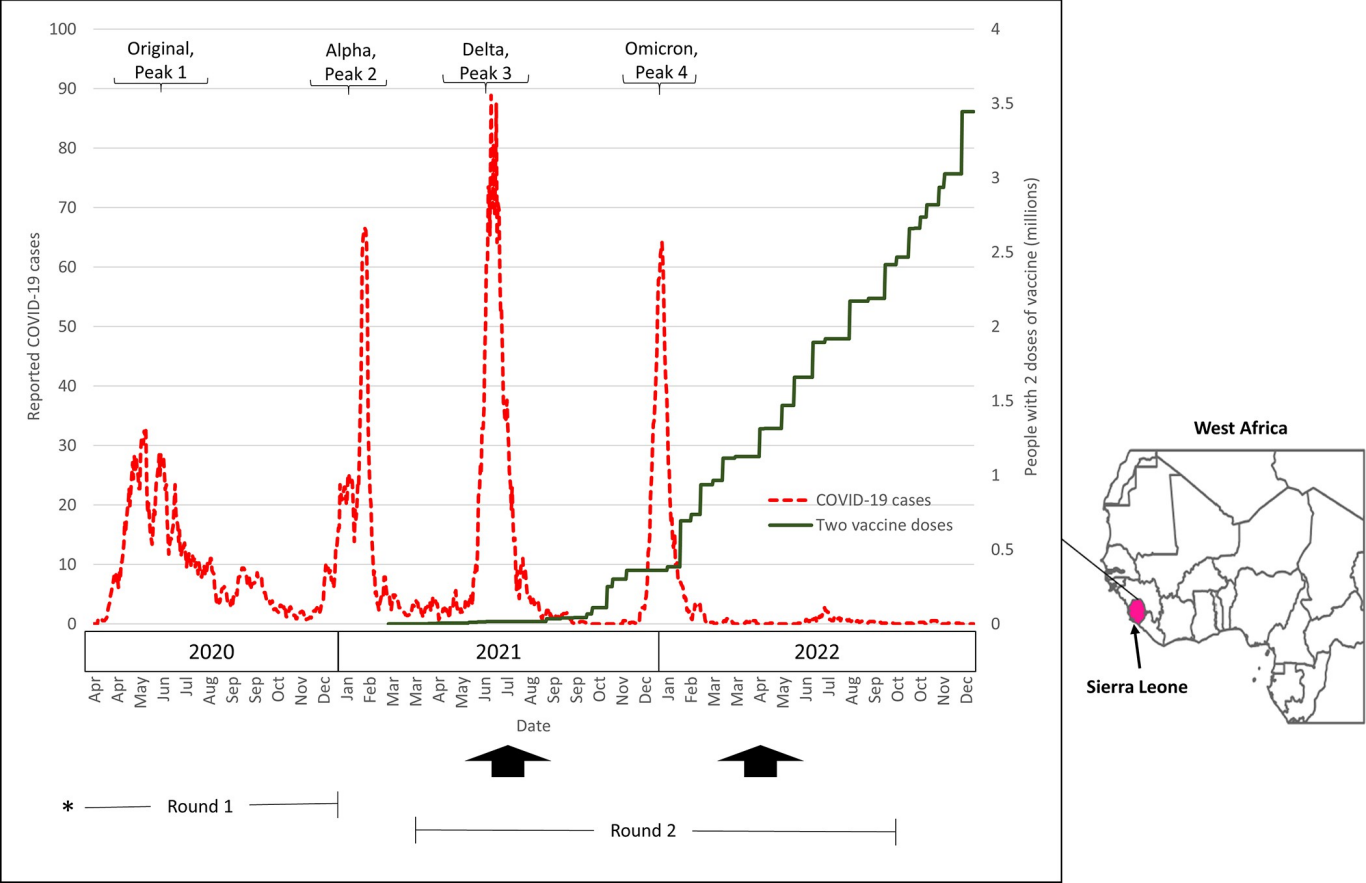
Timeline of reported COVID infections and cumulative number of people who received two SARS-CoV-2 vaccine doses in Sierra Leone. Data sources and indicator descriptions: Our World In Data: COVID infections, 7-day rolling averages, and total number of people who received the second dose of an authorized 2-dose vaccine; *duration of HEAL-SL fieldwork: May 2019 to January 2021 (Round 1), April 2021 to October 2022 (Round 2); vertical arrows represent serosurvey periods.

Sierra Leone had four major PCR-confirmed COVID peaks between 2020 and 2022: 1) May-August 2020 (Original variant of the SARS-CoV-2 virus), 2) January-February 2021 (mostly Alpha variant), 3) June-July 2021 (Delta variant), and 4) December 2021-January 2022 (Omicron variant; Fig 1). We conducted two serosurveys among 4,160 adults in Bo district, using the 41 Bo sampling units (drawn randomly from the 2016 census) in the Healthy Sierra Leone (HEAL-SL) project, a nationally representative mortality study [5]. Data collection teams (total of 17 field staff) went to all households in these units to enroll two adults per household, implemented a brief questionnaire on general health, and collected dried blood spots (DBS). The first DBS survey was done in July 2021, during the Delta wave (which was also during the rainy season when malaria transmission peaks) and the second round was in April 2022, including a subset of those tested in the first survey.

### SARS-CoV-2 seroincidence

We defined SARS-CoV-2 incidence based on the full-length spike protein of SARS-CoV-2 and its receptor-binding domain (RBD) using a highly sensitive and specific chemiluminescence assay [4]. There was little overlap of RBD or spike seropositivity with malaria status among 43 DBS samples collected from a study in 2017, while over a quarter of the archived 2017 samples tested positive to SARS-CoV-2 nucleocapsid protein (with a greater proportion among PCR-confirmed negative malaria patients (see footnote in Table 1). The low specificity of SARS-CoV-2 nucleocapsid is reflected in other African settings stemming from cross-reactivity of antibodies from previous exposure to other coronaviruses and infectious diseases such as malaria, which can interfere with the specificity of the current COVID-19 tests [6–8]. Thus, using the strictest definition is dual positivity to spike and RBD, cumulative seroincidence rose from 69% among the 224 adults (drawn randomly from the cohort of 4,160 adults) during the Delta wave in July 2021 to 84% among the 114 adults re-tested during the Omicron wave in April 2022 (Table 1). Using a less strict definition of infection of seropositivity to either RBD or spike, seroincidence rose from 91% to 98%, suggesting that nearly all adults had become infected by July 2021. About a fifth of the adults who were tested in both rounds and were seronegative during the Delta wave became seropositive by the Omicron wave, and close to two-thirds maintained seropositivity. About one in six reverted to seronegative.

**Table 1:** SARS-CoV-2 seroincidence by age, sex, and malaria positivity in Bo district, Sierra Leone during the pandemic (2021 and 2022) and pre-pandemic (2017).

Assay, characteristics, number sampled (male, female)	Delta wave: July 2021 N = 224 (94/130)	Omicron wave: April 2022 N = 114 (46/68)
RBD and Spike positive	70% (157)	84% (96)
Sex^†^		
Male	72% (94)	80% (46)
Female	69% (130)	87% (68)
Age		
18–29 years	69% (116)	85% (56)
30–44 years	71% (54)	83% (24)
45–59 years	80% (38)	80% (25)
60+ years	51% (16)	89% (9)
Either RBD or Spike positive	92% (207)	98% (112)
Nucleocapsid	68% (153)	74% (84)
**Neutralizing Antibodies** (N = 224, 94 male, 130 female)*	
Neutralizing>20%	43% (97)	
**Seroconversion between Delta and Omicron waves**^‡^ (N = 114, 46 males, 68 females)	
No change		64% (73)
Became positive		21% (24)
Became negative		15% (17)

^†^Positivity by sex and age are for receptor binding domain (RBD) and full-length spike proteins

^‡^seroconversion for RBD or spike proteins; out of 43 archived plasma samples from 2017, 27 were malaria negative (15 male, 12 female) and 33% of these (9/27) were positive for nucleocapsid only, 16 were malaria positive (5 male, 11 female) and 6% and 12% of these were positive for RBD or Spike (1/16) and nucleocapsid (2/16), respectively.

**Fig 2:**
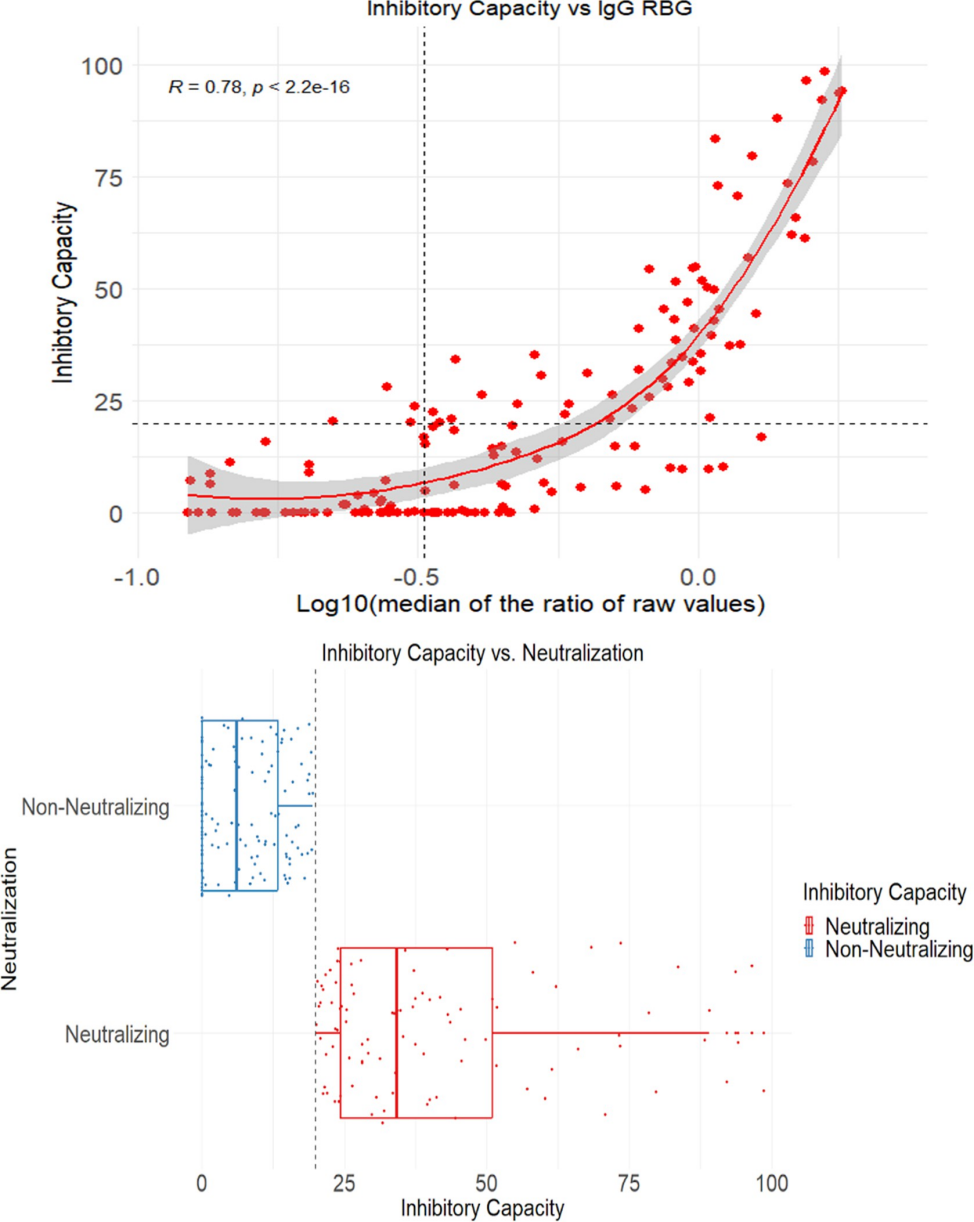
Correlation of titers of receptor binding domain (RBD) assay with neutralizing antibody (top panel) and Relative ratio (RR) by age and distributions of neutralizing and non-neutralizing antibody (bottom panel), in Bo district, Sierra Leone 2021 and 2022. The top panel illustrates the relationship between inhibitory capacity and the log 10 median of the ratio of raw values for the RBD IgG antibody response, based on 2.5 ul/well sample dilution. A cut off of 20% inhibitory capacity separates neutralizing from non-neutralizing antibodies, and a log10(0.324) cut-off separates positive from negative IgG RBD antibody responses. The relationship is fit using locally estimated scatterplot smoothing and assessed using a Pearson correlation coefficient. The bottom panel illustrates the inhibitory capacity in relation to neutralizing ability.

There were no important differences in RBD and spike positivity between men and women. The levels of RBD and spike antibodies in both rounds combined were generally independent of age, suggestive of widespread community transmission (S1 Fig). The majority (around 70%) of the tested population were below age 45 years. Among adults aged 60 years or above, cumulative seroincidence to RBD and spike during the Delta wave was lower but rose to nearly 100% during the Omicron wave. Among 224 adults tested during the Delta wave, 43% showed high levels of neutralizing antibodies. Neutralizing antibody titers were highly correlated with anti-RBD levels (Fig 2). This suggests that SARS-CoV-2 infection led to antibody responses sufficiently strong to induce neutralizing antibodies.

### Excess mortality for all-cause mortality

**Fig 3:**
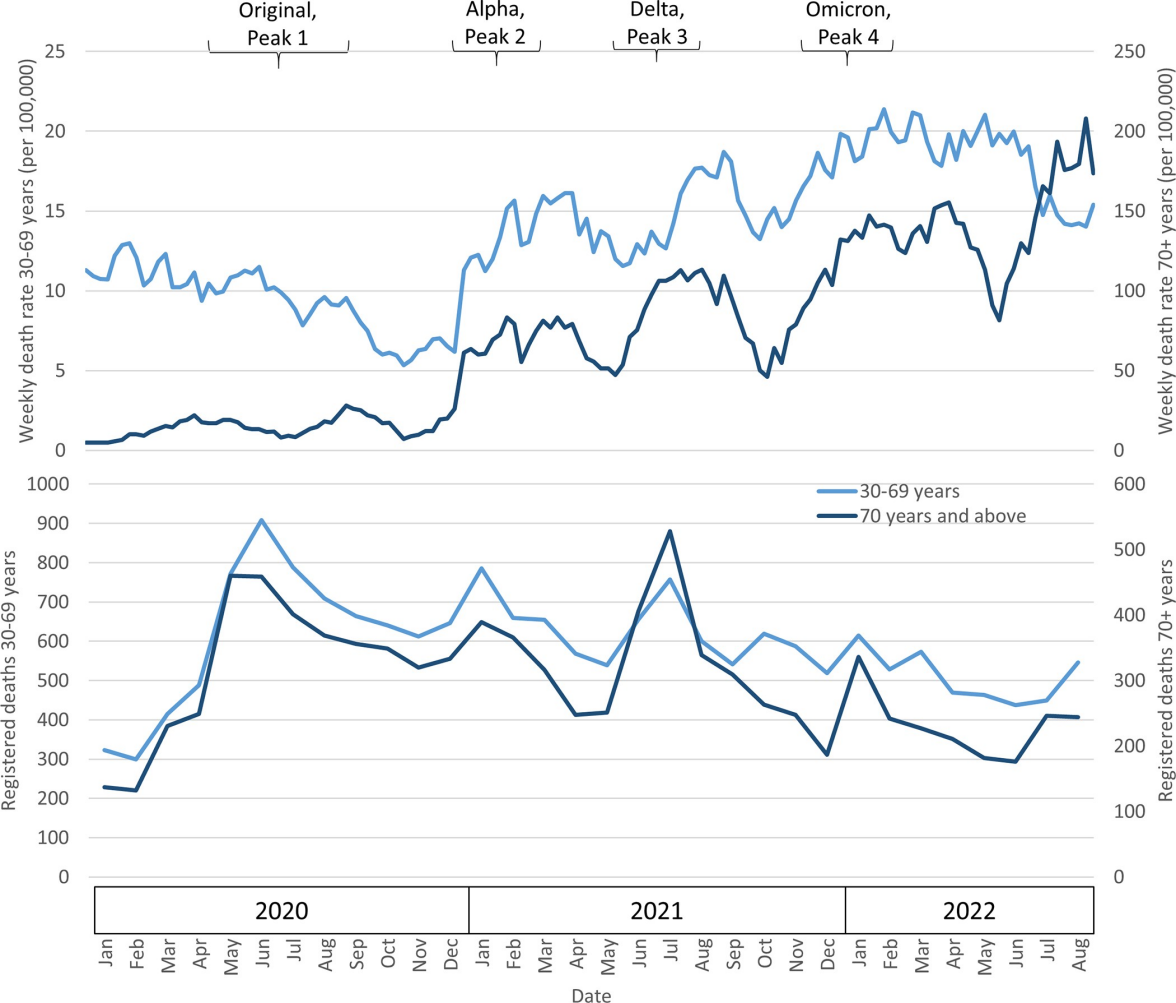
Age-specific mortality rate per 100,000 study population in Sierra Leone 2020–2022 from HEAL-SL (top panel) and registered deaths from NCRA (bottom panel). Data sources: Healthy Sierra Leone (HEAL-SL) rounds 1 and 2, National Civil Registration Authority (NCRA); mortality rates from HEAL-SL (2-month rolling averages) were calculated by summing the age-specific study deaths by week and dividing by the total study population enumerated in the same age group during the week of the reported deaths and afterwards. Total registered deaths from NCRA (both sexes): 19,277 in 2020 (7264 at 30–69 years, 3796 at ≥70 years), 19,114 in 2021 (7487 at 30–69 years, 3850 at ≥70 years) and 15,507 in 2022 (6378 at 30–69 years, 2834 at ≥70 years).

HEAL-SL’s nationally-representative mortality study covering 5% of randomly-selected households showed detectable increases in adult death rates (per 100,000 enumerated population) at ages 30–69 years and ≥70 years during the Alpha, Delta and Omicron waves, but less so after the original wave (Fig 3). The background mortality rates were, as expected, notably higher at ≥70 years but amplified further during the SAR-CoV-2 viral waves. The nascent Sierra Leone National Civil Registration Authority (NCRA) registered deaths covering less than a quarter of expected deaths in the country also showed notable peaks in the absolute number of deaths at ages 30–69 and ≥70 years for each of the viral waves. The peak that coincided with the Delta wave (June/July 2021) was especially pronounced for the ≥70 age group. The observed NCRA increases in the first half of 2020 coincided with a considerable expansion of death registration efforts, and thus may not represent solely viral wave-induced increases in mortality.

Because the HEAL-SL survey started in 2019 and did not include deaths ≥70 years during the first round (including deaths from 2018–2020), we lacked reliable historic data to compare excess deaths from all causes comparing the viral waves to pre-pandemic periods. Instead, we contrasted the average weekly death rate during the 22 viral peak weeks (Alpha, Delta and Omicron waves) between 2021 and 2022 to the average death rate during the 85 weeks in-between that were the “non-peaks” (Table 2). We estimated relative peak: non-peak mortality risks using Poisson regression (adjusted for each wave and temporality using week number), which resulted in a 22% excess at ages 30–69 years (RR 1.22; 95% CI 0.93, 1.61) and 70% excess at ages ≥70 years (RR 1.70; 1.23, 2.35). At ages 30–69 years, the peak: non-peak mortality risks were similar by sex, region, and urban/rural residence. The NCRA data showed a similar pattern of peak: non-peak mortality risks, based on logistic regression, showing a 10% excess at ages 30–69 years (RR 1.10; 1.05, 1.14) and a 22% excess at ≥70 years (RR 1.22; 1.16, 1.28). Logistic regression analyses comparing mortality rates in those under age 30 to the older age groups revealed a significant excess in ≥70 years age group (RR 1.43; 1.12,1.84; adjusting for linear and quadratic trends) but no excess in the 30–69 year age group (RR 0.98; 0.81, 1.19).

**Table 2:** Excess mortality in Sierra Leone comparing weekly death rates (per 100,000 population) from HEAL-SL and monthly death counts from NCRA during COVID-19 peak and non-peak periods by age, sex, region, and residence type.

	Average weekly death rates per 100,000 (number of deaths) *								Excess mortality RR ^†^	
	Alpha peak 2 (Jan-Feb 21)		Delta peak 3 (Jun-Jul 21)		Omicron peak 4 (Dec 21-Jan 22)		Non-peaks^‡^		Total peak vs non-peak	Poisson regression (95% CI)
**HEAL-SL (by age)**										
30–69 years	15	(96)	14	(85)	18	(62)	13	(657)	1.23	1.22 (0.93,1.61)
≥70 years	85	(40)	109	(50)	144	(39)	75	(305)	1.44	1.70 (1.23,2.35)
**HEAL-SL (30–69 years)**										
Sex										
Male	16	(50)	16	(48)	22	(37)	15	(382)	1.18	1.19 (0.89,1.58)
Female	14	(46)	12	(37)	14	(25)	10	(275)	1.29	1.29 (0.93,1.80)
Region										
Western	36	(46)	35	(37)	42	(25)	31	(275)	1.21	1.14 (0.74,1.74)
Rest of country	16	(78)	14	(71)	18	(51)	13	(550)	1.23	1.22 (0.92,1.62)
Residence										
Urban	16	(37)	15	(31)	20	(24)	13	(237)	1.27	1.28 (0.97,1.69)
Rural	15	(59)	14	(54)	17	(38)	12	(420)	1.20	1.21 (0.88,1.65)
	**Average monthly death counts (number of deaths)**									
**NCRA (by age)**										
30–69 years	723	(1445)	707	(1413)	567	(1134)	563	(10140)	1.18	1.10 (1.05,1.14)
≥70 years	378	(755)	467	(934)	262	(523)	270	(4860)	1.37	1.22 (1.16,1.28)

Healthy Sierra Leone (HEAL-SL) weekly data from Round 2 (2019–2022); National Civil Registration Authority (NCRA) monthly data

*weekly mortality rates were calculated by summing the age-specific study deaths by week and dividing by the total study population enumerated in the same age group during the week of the reported death and afterwards

^**†**^excess mortality rate ratios (RR) calculated as the total deaths per 100,000 divided by total weeks (for HEAL-SL) or total monthly death counts divided by total months (for NCRA) for peak and non-peak periods; excess mortality RR and 95% confidence intervals (CI) were also estimated using Poisson regression (for HEAL-SL) or logistic regression (for NCRA)

^**‡**^peaks defined as 7-day average of 8 new infections or more for at least 2 weeks, these analyses included peak 2 (1 Jan 2021 to 13 February 2021, 8 weeks), peak 3 (6 June 2021 to 23 July 2021, 8 weeks), peak 4 (20 December 2021 to 22 January 2022, 6 weeks); non-peaks defined as time periods occurring between week 34 of 2020 (August) and week 36 of 2022 (September), excluding peak periods (total 85 weeks).

Use of weekly averages as compared to the Poisson regression results yielded similar but slightly greater excesses. A potential limitation of this peak versus non-peak analysis approach is the possibility of a variable time lag from infection to death. This may result in COVID-19 deaths from a wave of infection that continue after the peak of the wave, meaning that the non-peak periods could also include several COVID-related deaths, and thus bias the estimates of excess deaths towards zero. To further investigate this potential limitation, we performed a sensitivity analysis running the Poisson regression using peak periods extended by two weeks. For the 70+ age group we found a slightly smaller RR of 1.68 (1.26–2.24). For the 30–60 year age group we found an increased RR of 1.34 (1.07–1.67), with results for both age groups largely consistent with our main analyses.

SARS-CoV-2 causes few death in children, adolescents, or young adults. We did not observe increased death rates at these ages during the viral peaks. To explore if the pandemic interrupted health services in ways that could raise child mortality, we compared deaths in children under five occurring in health facilities, as part of a national reporting system (S2 Fig). The weekly patterns of child deaths showed a notable spike prior to the original viral wave, and prior to the Alpha wave, but there was no clear excess of childhood deaths with each viral wave.

### Excess mortality for cause-specific mortality

HEAL-SL, the source of cause-of-death information for the analyses, is based on dual, independent physicians coding of electronic verbal autopsies [5]. We observed modest excess risks in peak versus non-peak death rates due to conditions that were considered strongly or weakly associated with COVID (Table 3), particularly at ages ≥70 years. The most notable excess deaths were from respiratory infections (RR 1.97; 1.33, 2.93). Among the causes of death with possible COVID association, infections showed the strongest correlation with viral peaks (RR 1.37; 1.07, 1.75).

**Table 3:** Excess mortality in Sierra Leone at ages ≥30 years comparing weekly average death rates (per 100,000 population) and number of deaths during COVID peak and non-peak periods by age group and cause of death.

	Weekly average deaths per 100,000 (number of deaths)				Excess mortality RR (95%CI) ^ⱡ^
	Peaks		Non-peaks		
**COVID-associated**	**8.2**	**(139)**	**6.0**	**(336)**	**1.38 (1.14,1.69)**
Vascular	5.3	(90)	4.2	(234)	1.29 (1.01,1.64)
Respiratory	2.3	(39)	1.2	(66)	1.97 (1.33,2.93)
Fever and infection	0.6	(10)	0.6	(36)	0.93 (0.46,1.87)
**Possibly COVID associated**	**10.9**	**(184)**	**8.8**	**(496**)	**1.24 (1.05,1.47)**
Other infectious	5.4	(91)	3.9	(222)	1.37 (1.07,1.75)
Digestive	2.4	(41)	2.2	(124)	1.10 (0.78,1.57)
Malaria	1.8	(31)	1.5	(85)	1.22 (0.81,1.84)
Cancer	0.4	(7)	0.4	(25)	0.94 (0.41,2.16)
Maternal	0.2	(3)	0.2	(10)	1.00 (0.28,3.64)
Other causes	0.7	(11)	0.5	(30)	1.23 (0.61,2.45)
**Not COVID associated**	**2.3**	**(39)**	**1.9**	**(106)**	**1.23 (0.85,1.78)**
Road and transport accidents	0.7	(11)	0.6	(33)	1.11 (0.56,2.20)
Other causes	1.7	(28)	1.3	(73)	1.28 (0.83,1.98)
Unknown	0.6	(10)	0.4	(24)	1.39 (0.67,2.91)
**All-cause mortality**	**22.1**	**(372)**	**17.1**	**(962)**	**1.29 (1.15,1.46)**

Data source: Healthy Sierra Leone (HEAL-SL) round 2 which covered deaths between 2019 and 2022; ⱡexcess mortality rate ratio (RR) calculated as the total weekly death rate for COVID peaks 2, 3, and 4 (corresponding with the Alpha, Delta, and Omicron waves during 2021–2022) divided by the total rate for all non-peak periods (September 2020–2022), 95% confidence intervals (CI) based on Poisson regression; We classified the ICD-10 codes based on expert opinion, and using published studies in high-income countries that excess COVID deaths can occur from vascular disease [9, 10]. We also identified conditions clearly unassociated with infection (such as accidents). Results were similar if we examined only deaths where both physicians immediately agreed on the diagnosis or if we used just deaths occurring in facilities. ICD-10 codes by category, and in order of decreasing frequency of numbers of deaths: “Fever and infection” (L03, R50, K04, A41, L02, A49, L08, M00, N30); “Other infectious” (B99, A01, A16, A15, A09, B24, B19, A03, A18, G03, A05, A95, B09, B20, B22, B74, B02, B16, B23, B65, B90, N73); “Digestive” (K27, K74, K76, R19, K72, K40, K46, K70, B18, K56, K35, K92, K37, K63, K36, K42, K45, R10, R11, R13, R18); causes of death included in “Other causes–possibly associated”: Other genitourinary system diseases, Neuro-psychiatric, Nephritis and nephrosis, Selected vaccine preventable diseases; causes of death included in “Other causes–not associated”: Skin, Musculoskeletal, Sense organ, Oral, Sickle-cell, Nutritional, other injuries.

The HEAL-SL verbal autopsy data showed that a similar proportion of those ≥30 years dying during peak versus non-peak periods had a history of at least one chronic disease (including heart disease, diabetes, cancer, kidney or liver disease, stroke, asthma, chronic lung disease; S1 Table) with similar results for 30–69 or ≥70 years. Moreover, the proportions of deaths at ages ≥30 years with reported high blood pressure or alcohol use was similar during peak and non-peak weeks, with slightly lower smoking prevalence among those who died during the peak weeks.

### National estimates of excess mortality

Based on United Nations mortality estimates, Sierra Leone had approximately 750 deaths per week at ages >30 years during the three-year period of 2020–2022 (https://population.un.org/wpp/). Applying the age-specific excess mortality to the 22 viral weeks of excess deaths yields an estimate of about 6,900 (range 1,500 to 15,000) excess deaths at these ages from 2020–2022 (Table 4). This accounts for about 5.8% (95% CI 1.3%, 12.8%) of all deaths over the three years. The model-based estimates by WHO report 7,900 excess deaths in Sierra Leone over the two-year period of 2020–2021 [2]. The HEAL-SL estimate corresponds to an excess mortality rate per 100,000 of 262 (range 59 to 576) at ages ≥30 years.

**Table 4:** National estimates of annual excess deaths and excess mortality rates during the COVID pandemic in Sierra Leone (2020–2022).

Setting and age group	Average per 100,000 population	(Lower, upper bounds)
**Excess death rate per 100,000 by age** WHO estimate for Sierra Leone for 2020–2021, all ages	95	(6, 192)
Sierra Leone for 2020–2022, ages ≥30 years, present study	262	(59, 576)
**Total excess deaths**		
Total ≥30 years in Sierra Leone for 2020–2022 (three years), present study	6,900	(1,500, 15,000)
WHO estimate for Sierra Leone, all ages for 2020–2021 (two years)	7,900	(480, 15,700)
Excess as percent of deaths ≥30 years in Sierra Leone for 2020–2022	5.8%	(1.3%, 12.8%)

Calculations used national death estimates from the UN World Population Prospects for average of deaths in 2019, 2020, and 2021; excess proportions estimated using Poisson regression applied to 22 peak weeks: 0% and 61% for 30–69 years and 23% and 135% for 70 years and above. *WHO estimates for all of Africa [2].

## Discussion

Sierra Leone appears to have experienced widespread SARS-CoV-2 transmission, but relatively low levels of excess mortality from COVID. Cumulative seroincidence, even using the strictest definition of infection, was 69% by July 2021, rising to 84% by April 2022. The April 2022 serosurvey preceded any widespread vaccination coverage (approximately 14% of the total population), thus only a small proportion of second round participants may have had shown seropositivity from vaccination. About half of these infections also generated neutralizing antibody responses, which is a correlate of protection. Despite high levels of prior infection, the Omicron viral wave infected at least 20% of adults. Our study adds to earlier analyses documenting widespread SARS-CoV-2 transmission in Africa [11, 12]. Our seroincidence findings, as well as overviews of seroincidence in various African countries [11], are markedly higher than the low 4% seroincidence measured in March 2021 in one study [13]. The March 2021 study used a point of care test which can have uncertainties [6].

Despite very high and rapid transmission, excess death rates from COVID appear to have been low in Sierra Leone, and the deaths that did occur were mostly concentrated at older ages. During the peak viral weeks, excess mortality was 22% at ages 30–69 years and 70% at age ≥70 years. Observed excess deaths were notable for respiratory infections but did not differ greatly across specific causes that would, a priori, be strongly associated with COVID, nor among those with or without chronic disease risk factors.

There might be several explanations for Sierra Leone’s pattern of high seroincidence and low mortality, implying a lower infection-fatality rate than observed in much of the world outside Africa [14]. First, Sierra Leone’s young age structure would mean relatively fewer absolute deaths at older ages, during which COVID mortality is highest. Second, high background mortality could mask COVID mortality. At 2022 death rates, nearly 37% of Sierra Leoneans aged 30 can expect to die before age 70 from various conditions, including malaria [5]. Thus, even if infection-fatality rates were similar in Sierra Leone to high-income countries, high background mortality would reduce percentages of excess mortality. Apart from these interpretations, additional biological studies are required to shed light on the apparent heterogeneities in susceptibility in infection fatality rates. Third, the widespread infection, much of which generated neutralizing antibody levels, may protect against disease but not against re-infection. It is unknown why widespread Sierra Leone SARS-CoV-2 infection behaved similar to the effect of current mRNA or adenovirus-based vaccines. In various populations, two-doses of most SARS-Cov-2 vaccines generally have induced excellent protection against disease but less so against infection or re-infection [15, 16].

The low levels of observable disease and deaths in Sierra Leone are in marked contrast to other settings that also had widespread community transmission of SARS-CoV-2. In India widespread infection during the initial viral waves did not protect against a large increase in deaths in subsequent viral waves [17, 18]. The approximate 6% excess of deaths at ages 30 years or more from 2020 to 2022 in Sierra Leone is markedly lower than in India and South Africa (26% each), and Peru (49%) [26]. Sierra Leone’s results are broadly consistent with reported excess death rates from COVID in The Gambia [19], Namibia [20], and Sudan [21], but lower than in coastal Kenya at older ages [22], and South Africa at various ages [23, 24]. By contrast, the estimated excess death rates in Sierra Leone were notably higher than those reported in Zambia [25] and Madagascar [26].

While we did not observe any protective effects of the currently lower prevalence of diabetes, obesity, and other risk factors, there might be some unknown and genetic predictors of disease. Genetic causes seem less plausible as among African-Americans, COVID mortality and hospitalization rates are high, despite sharing common ancestral genes with West Africa, including any polymorphisms in the receptor gene for the ACE-2 protein, which is the key for the SARS-CoV-2 to enter cells [27]. Analyses of T-cell responses in Ghana, also in West Africa, suggest that prior malaria exposure may influence the T-cell mediated immune response to SARS-CoV-2 infection, leading to milder disease in malaria-endemic regions [28]. The highly sensitive and specific RBD or Spike protein antibody responses were unaffected by past malaria infection. However, our finding that nucleocapsid positivity was higher in archived malaria negative versus malaria positive adults is consistent with the Ghanaian result.

Our study has several strengths, including use of nationally representative mortality data from at least two sources, and robust highly sensitive and specific chemiluminescence and neutralizing antibody assays. However, Sierra Leone is not typical of the whole of Africa and there likely is marked regional variation in the correlates of infection and of COVID disease, as observed by the variation in excess mortality between South Africa, Lusaka, and other parts of the continent. The serosurvey was done in one district only, but the high prevalence and consistency of excess mortality results across various regions and demographic groups suggests that widespread transmission and infection occurred throughout Sierra Leone. Third, we cannot exclude recall bias in reporting deaths, which could mean missing deaths at older ages paired with digit preference for age of death [29, 30]. Verbal autopsy studies in other settings, however, have found recall of important events such as deaths to be reasonably reliable. Sierra Leone suffered a major Ebola virus outbreak in 2016, and there might well be some stigma in reporting deaths, particularly early on during the COVID pandemic. Finally, the HEAL-SL as a new survey will have inconsistencies in establishing a stable population baseline, which affects the calculated death rates. For this reason, we relied on comparisons of peak weeks to inter-peak weeks, which should be less prone to initial under-registration of populations or deaths, though may be influenced by seasonality. Sierra Leone’s tropical climate may have introduced a seasonal factor that affected mortality from other infectious causes such as malaria. However, given that malaria transmission occurs throughout the year, we expected any resulting biases to be small. Our analyses using peaks and non-peaks risks under-estimating excess deaths if COVID deaths continue during non-peak periods. However, a sensitivity analysis extending the peak mortality periods by at least two weeks showed similar results. This conclusion is plausible, as access to hospital care is very low in Sierra Leone, and for those who are hospitalized, intubation services are nearly unavailable. Therefore, the interval from infection to death is likely close to that from infection to hospitalization in places where hospital care is readily available. As an additional check, we examined excess mortality models using monthly data from Botswana [31] using a model similar to the WHO method used by Msemburi et al (2023) [2], and found that the use of inter-wave deaths as the reference comparison was similar to the predicted trends from 2017 to 2020, prior to the pandemic. Thus, our use of inter-wave reference periods yields wider uncertainty ranges than pre and post pandemic comparisons but should not spuriously lower estimates of excess deaths.

In conclusion, Sierra Leone had widespread SARS-CoV-2 infection and transmission, but with little excess mortality. The specific disease pattern, with more infection than vaccination, might provide new insights or suggest new approaches. This includes the key question, which should be tested in randomized trials, if a single vaccine dose (or booster doses) targeted to older adults might provide sufficient additional protection against COVID disease [32, 33]. Despite widespread transmission, new variants did not arise out of Africa for several months after the first Omicron variant. Thus, Sierra Leone’s hybrid immunity from infection and vaccination took a very different time course than in high-income settings (which relied far more on vaccination) [15]. Additional biological and epidemiological studies in Africa, including in malaria-endemic regions, on the correlates of SARS-CoV-2 infection and its sequelae are essential [34].

## Methods

### Sample collection

In Bo district, we conducted a household survey in 41 randomly selected areas (based on the 2016 Census and using the HEAL-SL sampling frame- details published in Carshon-Marsh et al., 2022 [5]) in July 2021. The HEAL-SL sampling frame is drawn from the 2016 census and provides representative survey of about 5% of all the homes in Sierra Leone, including in Bo district. This particular sub-study comprised adults in Bo City (around 4000 participants), rural (approximately 3000), and child participants (roughly 1000). Teams of two trained professionals collected the dried blood samples (DBS) at the residences of 4160 adults gathering anthropometric, health, and sociodemographic data. From these, we randomly selected, using random digit selection of the DBS samples, a total of 227 for SARS-CoV-2 antibody testing. Of these three were later shown to be duplicates, yielding 224 randomly selected adults used in the study. A subsequent field collection in April 2022 was undertaken to return to these original households and was able to re-collect 114 out of the initial 224 participants. The remaining 110 participants were not contactable based on a single household visit.

We incorporated an additional set of 43 pre-pandemic whole blood samples into our study, randomly selected from a prior research initiative conducted at Mercy Hospital Laboratory in Bo City, Bo District. The laboratory-based study included 182 adults with fever who had sought malaria testing between April 2017 and July 2018. These whole blood samples were initially stored within a temperature range of 4°C to 20°C until August of 2021, subsequently undergoing centrifugation at 4000 rpm for 10 minutes. Following this step, the lab technician transferred four distinct 50μl drops of plasma onto DBS Whatman 903 paper [35].

### IgG serology

After collection, each bar-coded DBS card was scanned, linked to the individual, and then enclosed within a Mylar pouch containing 1g of desiccant (designed specifically to keep humidity levels low in Sierra Leone’s environment). These DBS cards were stored at -20°C in Mercy Hospital Laboratory. The randomly selected DBS samples were transported to the Network Biology Collaborative Centre at Sinai Health in Toronto, where a high-throughput chemiluminescence ELISA was conducted, targeting the full-length spike trimer, spike RBD, and nucleocapsid antigens using IgG#5-HRP as the secondary antibody (all antigens and IgG#5-HRP were provided by the National Research Council (NRC) of Canada). Raw chemiluminescent values were normalized to a synthetic standard included on each assay plate (VHH72-Fc supplied by NRC for spike/RBD or an anti-nucleocapsid IgG Ab from Genscript, #A02039) to generate relative ratios. The determination of seropositivity for each of these antigens had been previously published: at the selected thresholds (relative ratios at a 1:4 dilution of DBS eluate– 0.482 for spike, 0.324 for RBD, and 0.642 for nucleocapsid) set at 99% specificity, the sensitivities are 98% to spike and RBD and 92% to nucleocapsid for samples tested in Canada [4]. Our criterion for a positive result was based on the detection of spike and RBD based on the highest sensitivity and specificity for SARS-CoV-2.

The University of Ottawa tested all 224 samples from the 2021 survey for neutralization. A snELISA was carried out to evaluate neutralizing activity against the SARS-CoV-2 S protein, detailed in Galipeau et.al (2021) [36]. Neutralizing activity was determined by calculating the inhibitory capacity against SARS-CoV-2 S proteins by dividing the average mean of the luminescence signal of the sample by that of the control. We defined a sample with an inhibitory capacity above 20% to have a neutralizing antibody response.

### COVID infection data

We obtained daily COVID case counts for Sierra Leone from coronavirus.app, which uses the same data as Our World in Data [37]. We also reviewed the websites of the Ministry of Health and Sanitation (MOHS), and its national COVID program.

### Mortality data

We obtained mortality data from three sources: Healthy Sierra Leone (HEAL-SL), the National Civil Registration Authority (NCRA), and the electronic Integrated Disease Surveillance and Response facility-based surveillance system (eIDSR). HEAL-SL is an ongoing community-based mortality study, which collects data at the household level on the number and causes of death at any age using verbal autopsy. Details of the study design and implementation are published elsewhere [5]. Briefly, HEAL-SL uses a sampling frame covering approximately five percent of the country’s population, including about 60,000 households from urban and rural census enumeration areas distributed across the country’s five regions and 16 districts. Multiple teams of four full-time trained surveyors collect data from adult household members in selected sampling units using an instrument with three modules: enumeration, verbal autopsy, and resampling. The electronic verbal autopsy (e-VA) uses the 2016 WHO Verbal Autopsy Standard tool for neonates, children, adults, and stillbirths. Each death is assigned an International Classification of Diseases (ICD-10) code as underlying cause of death based on review of the e-VA and narrative by two independent physicians specially trained on death certification and ICD coding of verbal autopsy. These physicians assign causes anonymously, with reconciliation of differences and blind adjudication by a senior physician. The current analysis includes data from the second round of HEAL-SL, which were collected between April 2021 and October 2022, and include deaths at all ages occurring from 2019 to 2022. The lead implementer for HEAL-SL is the MOHS with support from Njala University in Bo and the University of Toronto Centre for Global Health Research.

The Sierra Leone NCRA data includes registered deaths by age and sex from 2019 (when the department began operations) to 2022. Data are collected on a monthly basis by region, district, sex, and age; including information on registration status (current, late, or delayed registration) the location of death (health facility or outside of heath facility), type of death (maternal or other), and whether a death certificate was issued. It does not register cause of death.

Facility-based data on mortality and clinical malaria cases for children under 5 years were obtained from the Sierra Leone eIDSR. The eIDSR system uses treatment and morbidity registers to collect data from all levels of health facility from large district hospitals to small peripheral health units across the country. Mortality data from these registers are summarized at the district level and aggregated into two major age groups (under five years, five years and above) and analyzed weekly (for the IDSR weekly report) and monthly (for the district HMIS quarterly report). District-level data are then fed into the national reporting system, which is managed using the District Health Information Software version 2 platform. Sierra Leone adopted technical guidelines to implement the IDSR framework developed by the WHO Regional Office for Africa (https://www.afro.who.int/publications/technical-guidelines-integrated-disease-surveillance-and-response-african-region-third) and since 2008, has established a comprehensive public health surveillance and response system for priority diseases, conditions, and events at all levels of the health system.

### Statistical analyses

We defined COVID peaks as any period of at least two weeks when the seven-day average number of new infections reported by the Government of Sierra Leone or the various global monitors were eight or more. Using this definition, four peaks were identified between 2020 and 2022: 1) 2 May 2020 to 15 August 2020 (16 weeks), 2) 30 December 2020 to 13 February 2021 (eight weeks), 3) 6 June 2021 to 23 July 2021 (eight weeks), and 4) 20 December 2021 to 22 January 2022 (six weeks). This analysis included peaks 2, 3, and 4, corresponding with the data collection period for round 2 of the HEAL-SL survey. We defined COVID-19 non-peak periods (troughs) as the periods between peaks; therefore, troughs one through four were 18, 15, 20, and 32 weeks in duration, respectively.

We calculated excess mortality, comparing peak to non-peak periods, as the average weekly death rate (per 100,000 population) for peaks divided by the average rate for non-peaks. Population denominators for each age group (30–69 years, 70 years and above) or demographic stratum (sex, urban or rural residence, region) of interest consisted of the weekly sum of enumerated household members (in the corresponding age group or stratum) during the week of the reported death and subsequent weeks until the end of the observation period (September 2022).

We analysed NCRA death registration data as monthly counts after converting the definitions of peaks and non-peaks from weeks to months, giving a total of 10 peak months and 22 non-peak months. We compared monthly death counts for peaks to monthly counts for non-peaks to estimate the mortality ratio for middle ages (30–69 years) and older ages (≥70 years) relative to deaths under 30 years of age. To estimate excess mortality by cause of death, we grouped physician-coded deaths according to a standard death classification system based on the (WHO) Global Health Estimates and adapted from the Indian National Burden Estimates [38, 39]. This comprises 45 distinct groups of causes drawn from the entire range of 474 unique ICD-10 codes captured in the study. We used expert opinion to categorize each death’s likely association with COVID. We compared peak and non-peak death rates across these broad cause of death categories, including: 1) COVID-associated: vascular, respiratory, fever and infection; 2) possibly COVID-associated: digestive, malaria, cancer, maternal, other infectious, other (other genitourinary system diseases, selected vaccine preventable diseases, nephritis and nephrosis, neuro-psychiatric conditions); 3) not COVID-associated: road traffic/other transport accidents and other injuries, sickle-cell disorders, and other skin, musculoskeletal, sense organ, or oral conditions; and 4) unknown causes. We used verbal autopsy data from all deaths at 30 years and above to calculate the proportions of deaths with a history of at least one chronic disease (including heart disease, diabetes, cancer, kidney or liver disease, stroke, asthma, or chronic lung disease) or risk factor (including high blood pressure, tobacco or alcohol use). These proportions were compared descriptively across COVID-19 peak and non-peak periods overall for both sexes.

We applied Poisson regression to estimate excess mortality using HEAL-SL data. We applied separate Poisson regression models for age groups 30–69 years and ≥70 years using HEAL-SL weekly death counts and person-week time from September 2020 to October 2022. The model also included week number as a covariate to identify trends in mortality. For all Poisson analyses, a robust variance estimator considered any extra-Poisson variation in the weekly counts. For the NCRA data no denominator data were available and–assuming negligible excess mortality in the under 30s –we used logistic regression to compare monthly deaths among the two older age groups (30–69 years and ≥70 years) relative to the under 30 group, during peaks (vs. non-peaks) from September 2020 onwards. Each model also included month number and its square ("month^2^") as covariates to adjust for any trends in mortality. Essentially, estimating whether the probability of a death being in the older age group depended on whether it occurred during a peak or non-peak month. The odds ratio (OR) from these analyses are a direct estimate of the incident rate ratio of peaks vs non-peaks among the older age group. Excess mortality analyses were conducted using STATA (version 15.1) and SPSS (version 22). Seroincidence analyses were conducted using R (version 4.2.1).

### Ethics approval

The HEAL-SL was approved by the Sierra Leone Ethics and Scientific Review Committee, Ministry of Health and Sanitation in March 2019 with an amendment approval in March 2022 to include the serosurvey. Informed written consent was obtained by all participants, including the parents/guardians of child participants.

## Supporting information

S1 Checklist (DOCX)

S1 Fig Distributions of antibodies to RBD by age (top panel) and round of serosurvey (bottom panel) in Bo district, Sierra Leone 2021 and 2022. Top panel illustrates the relationship between age group and the log 10 median of the ratio of raw values for the RBD IgG antibody response, based on 2.5 μl/well sample dilution. Dashed line represents the cut-off for seropositive RBD IgG status. Bottom panel illustrates the relationship between round of collection and the log 10 median of the ratio of raw values for the RBD IgG antibody response, based on 2.5 μl/well sample dilution. Dashed line represents the cut-off for seropositive RBD IgG status. Round 1 occurred in July 2021, and round 2 in April 2022.
(PDF)

S2 Fig Health facility deaths and clinical malaria by week for children under 5 years in Sierra Leone 2020–2022. Data source: electronic Integrated Disease Surveillance and Response (eIDSR); deaths and clinical malaria cases are presented as 8-week rolling averages; clinical malaria is defined as the total number of clinically diagnosed malaria cases not yet rapid test or laboratory confirmed; total deaths <5 years from eIDSR (both sexes): 56 in 2020, 86 in 2021 and 51 in 2022; total clinical malaria cases <5 years from eIDSR (both sexes): 1447415 in 2020, 1507575 in 2021 and 1452362 in 2022.
(PDF)

S1 Table Proportion of all deaths 30+ years with a history of any chronic disease or risk factor during COVID-19 peak and non-peak periods in Sierra Leone. Data source: Healthy Sierra Leone (HEAL-SL) round 2, which covered deaths between 2019 and 2022; 3 out of 4 COVID peak periods were included in the analysis corresponding with the Alpha, Delta, and Omicron waves during 2021 and 2022; non-peak weeks ranged from week 34 of 2020 (August) and week 36 of 2022 (September) excluding peak periods; total deaths at 30–69 years: 243 during peak weeks, 657 during non-peak weeks; total deaths at ≥70 years: 129 during peak weeks, 305 during non-peak weeks; *Other chronic diseases includes kidney disease, cancer, chronic lung disease.
(PDF)
