# Supplementary material for: High SARS-CoV-2 seroincidence but low excess COVID mortality in Sierra Leone in 2020–2022
Source: PLOS Glob Public Health. 2024 Sep 10;4(9):e0003411. doi: 10.1371/journal.pgph.0003411 (PMC11386415; doi:10.1371/journal.pgph.0003411)
Supplement: S1 Fig — Top panel illustrates the relationship between age group and the log 10 median of the ratio of raw values for the RBD IgG antibody response, based on 2.5 μl/well sample dilution. Dashed line represents the cut-off for seropositive RBD IgG status. Bottom panel illustrates the relationship between round of collection and the log 10 median of the ratio of raw values for the RBD IgG antibody response, based on 2.5 μl/well sample dilution. Dashed line represents the cut-off for seropositive RBD IgG status. Round 1 occurred in July 2021, and round 2 in April 2022. (PDF) [file pgph.0003411.s002.pdf]

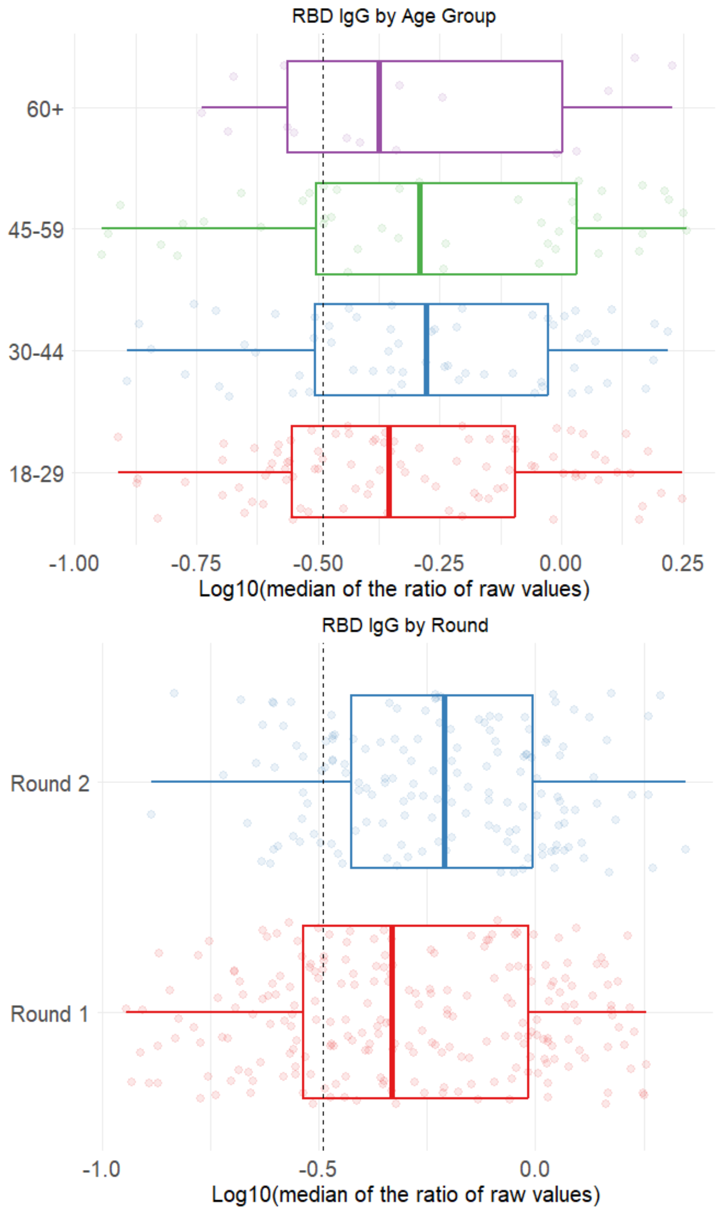

S1 Fig: Distributions of antibodies to RBD by age (top panel) and round of serosurvey (bottom panel) in Bo district, Sierra Leone 2021 and 2022

Top panel illustrates the relationship between age group and the log 10 median of the ratio of raw values for the RBD IgG antibody response, based on 2.5 ul/well sample dilution. Dashed line represents the cut-off for seropositive RBD IgG status. Bottom panel illustrates the relationship between round of collection and the log 10 median of the ratio of raw values for the RBD IgG antibody response, based on 2.5 ul/well sample dilution. Dashed line represents the cut-off for seropositive RBD IgG status. Round 1 occurred in July 2021, and round 2 in April 2022.
