# Supplementary material for: High SARS-CoV-2 seroincidence but low excess COVID mortality in Sierra Leone in 2020–2022
Source: PLOS Glob Public Health. 2024 Sep 10;4(9):e0003411. doi: 10.1371/journal.pgph.0003411 (PMC11386415; doi:10.1371/journal.pgph.0003411)
Supplement: S2 Fig — Data source: electronic Integrated Disease Surveillance and Response (eIDSR); deaths and clinical malaria cases are presented as 8-week rolling averages; clinical malaria is defined as the total number of clinically diagnosed malaria cases not yet rapid test or laboratory confirmed; total deaths <5 years from eIDSR (both sexes): 56 in 2020, 86 in 2021 and 51 in 2022; total clinical malaria cases <5 years from eIDSR (both sexes): 1447415 in 2020, 1507575 in 2021 and 1452362 in 2022. (PDF) [file pgph.0003411.s003.pdf]

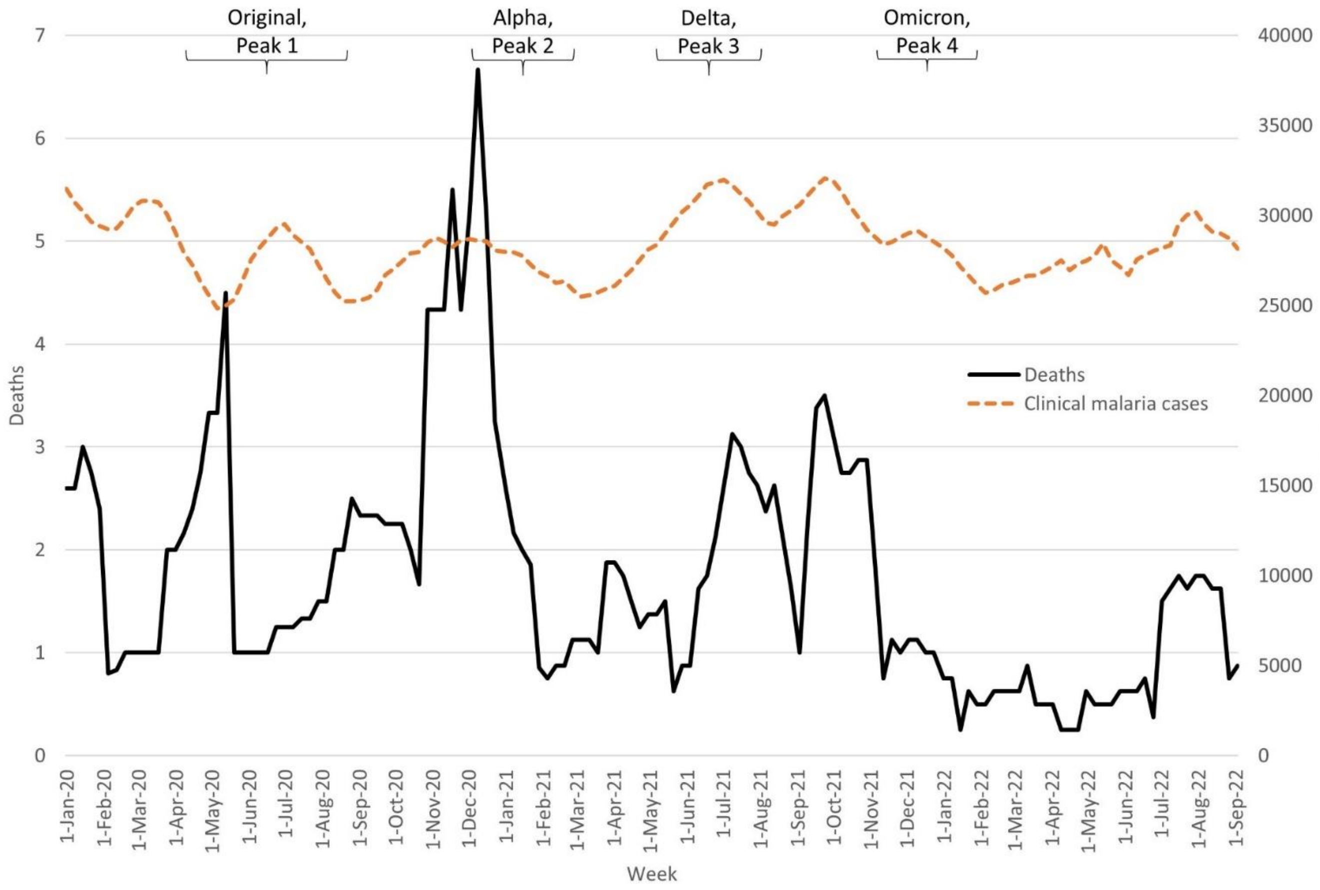

S2 Fig: Health facility deaths and clinical malaria by week for children under 5 years in Sierra Leone 2020-2022

Data source: electronic Integrated Disease Surveillance and Response (eIDSR); deaths and clinical malaria cases are presented as 8-week rolling averages; clinical malaria is defined as the total number of clinically diagnosed malaria cases not yet rapid test or laboratory confirmed; total deaths <5 years from eIDSR (both sexes): 56 in 2020, 86 in 2021 and 51 in 2022; total clinical malaria cases <5 years from eIDSR (both sexes): 1447415 in 2020, 1507575 in 2021 and 1452362 in 2022.
