# Supplementary material for: High SARS-CoV-2 seroincidence but low excess COVID mortality in Sierra Leone in 2020–2022
Source: PLOS Glob Public Health. 2024 Sep 10;4(9):e0003411. doi: 10.1371/journal.pgph.0003411 (PMC11386415; doi:10.1371/journal.pgph.0003411)
Supplement: S1 Table — Data source: Healthy Sierra Leone (HEAL-SL) round 2, which covered deaths between 2019 and 2022; 3 out of 4 COVID peak periods were included in the analysis corresponding with the Alpha, Delta, and Omicron waves during 2021 and 2022; non-peak weeks ranged from week 34 of 2020 (August) and week 36 of 2022 (September) excluding peak periods; total deaths at 30–69 years: 243 during peak weeks, 657 during non-peak weeks; total deaths at ≥70 years: 129 during peak weeks, 305 during non-peak weeks; *Other chronic diseases includes kidney disease, cancer, chronic lung disease. (PDF) [file pgph.0003411.s004.pdf]

S1 Table: Proportion of all deaths 30+ years with a history of any chronic disease or risk factor during COVID-19 peak and non-peak periods in Sierra Leone

	Proportion of peak deaths (number)		Proportion of non-peak deaths (number)	
**Chronic disease**	**13.7%**	**(51)**	**15.3%**	**(147)**
Diabetes	3.2%	(12)	3.2%	(31)
Stroke	2.4%	(9)	1.6%	(15)
Asthma	2.4%	(9)	2.6%	(25)
Liver disease	1.9%	(7)	4.4%	(42)
Heart disease	1.1%	(4)	1.8%	(17)
Other*	2.7%	(10)	1.8%	(17)
**Risk factors**	**44.4%**	**(165)**	**47.3%**	**(455)**
Smoking	14.0%	(52)	17.9%	(172)
Alcohol use	11.0%	(41)	11.5%	(111)
High blood pressure	19.4%	(72)	17.9%	(172)

Data source: Healthy Sierra Leone (HEAL-SL) round 2, which covered deaths between 2019 and 2022; 3 out of 4 COVID peak periods were included in the analysis corresponding with the Alpha, Delta, and Omicron waves during 2021 and 2022; non-peak weeks ranged from week 34 of 2020 (August) and week 36 of 2022 (September) excluding peak periods; total deaths at 30-69 years: 243 during peak weeks, 657 during non-peak weeks; total deaths at ≥70 years: 129 during peak weeks, 305 during non-peak weeks; *Other chronic diseases includes kidney disease, cancer, chronic lung disease.
